# Polyhydroxyalkanoate Copolymer Production by Recombinant *Ralstonia eutropha* Strain 1F2 from Fructose or Carbon Dioxide as Sole Carbon Source

**DOI:** 10.3390/bioengineering11050455

**Published:** 2024-05-02

**Authors:** Chih-Ting Wang, Ramamoorthi M Sivashankari, Yuki Miyahara, Takeharu Tsuge

**Affiliations:** Department of Materials Science and Engineering, Tokyo Institute of Technology, 4259 Nagatsuta, Midori-ku, Yokohama 226-8502, Japan

**Keywords:** polyhydroxyalkanoate, autotroph, α-methylated monomer, 3-hydroxy-2-methylpropionate, chiral configuration

## Abstract

*Ralstonia eutropha* strain H16 is a chemoautotrophic bacterium that oxidizes hydrogen and accumulates poly[(*R*)-3-hydroxybutyrate] [P(3HB)], a prominent polyhydroxyalkanoate (PHA), within its cell. *R. eutropha* utilizes fructose or CO_2_ as its sole carbon source for this process. A PHA-negative mutant of strain H16, known as *R. eutropha* strain PHB^−^4, cannot produce PHA. Strain 1F2, derived from strain PHB^−^4, is a leucine analog-resistant mutant. Remarkably, the recombinant 1F2 strain exhibits the capacity to synthesize 3HB-based PHA copolymers containing 3-hydroxyvalerate (3HV) and 3-hydroxy-4-methyvalerate (3H4MV) comonomer units from fructose or CO_2._ This ability is conferred by the expression of a broad substrate-specific PHA synthase and tolerance to feedback inhibition of branched amino acids. However, the total amount of comonomer units incorporated into PHA was up to around 5 mol%. In this study, strain 1F2 underwent genetic engineering to augment the comonomer supply incorporated into PHA. This enhancement involved several modifications, including the additional expression of the broad substrate-specific 3-ketothiolase gene (*bktB*), the heterologous expression of the 2-ketoacid decarboxylase gene (*kivd*), and the phenylacetaldehyde dehydrogenase gene (*padA*). Furthermore, the genome of strain 1F2 was altered through the deletion of the 3-hydroxyacyl-CoA dehydrogenase gene (*hbdH*). The introduction of *bktB-kivd-padA* resulted in increased 3HV incorporation, reaching 13.9 mol% from fructose and 6.4 mol% from CO_2_. Additionally, the *hbdH* deletion resulted in the production of PHA copolymers containing (*S*)-3-hydroxy-2-methylpropionate (3H2MP). Interestingly, *hbdH* deletion increased the weight-average molecular weight of the PHA to over 3.0 × 10^6^ on fructose. Thus, it demonstrates the positive effects of *hbdH* deletion on the copolymer composition and molecular weight of PHA.

## 1. Introduction

Petroleum-based plastics have become indispensable in human life, serving a multitude of purposes [1,2,3]. However, their widespread utilization poses significant environmental and health hazards [1,2,3]. Indeed, petroleum-based plastics contribute significantly to greenhouse gas emissions, including CO_2_, throughout their manufacturing and processing stages, intensifying the challenges of global warming and climate instability [4,5]. Furthermore, their resistance to degradation and challenges in recycling further exacerbate resource depletion and pollution concerns [6]. Consequently, there is an urgent need to develop biodegradable renewable alternatives [7].

Polyhydroxyalkanoate (PHA) is a microbial polyester recognized for its exceptional biodegradability, which makes it a promising environmentally friendly plastic [1,2,3,4,5,6]. The structure and characteristics of PHA vary according to their monomers, with poly[(*R*)-3-hydroxybutyrate] [P(3HB)] being the most common and basic form. However, P(3HB) faces challenges, such as high crystallinity and low elasticity, which limit its practical applications. To address these limitations, various PHA copolymers have been developed by copolymerizing 3HB with other monomers such as 3-hydroxyvalerate (3HV), 3-hydroxy-4-methylvalerate (3H4MV), and 3-hydroxyhexanoate (3HHx) [1,8]. 3HB-based copolymers enhance the overall material properties of PHA, surpassing the limitations associated with P(3HB) alone [1,8]. PHAs incorporating α-methylated monomers, namely 3-hydroxy-2-methylbutyrate (3H2MB), 3-hydroxy-2-methylpropionate (3H2MP), 3-hydroxypivalate (3HPi), and 3-hydroxy-2-methylvalerate (3H2MV), epitomize pioneering bio-based materials. These monomers confer superior crystallization behavior to polymers [9,10] and exhibit high thermal stability [11,12], rendering them tough.

*Ralstonia eutropha* H16 (also known as *Cupriavidus necator* H16) is a chemoautotrophic bacterium capable of oxidizing hydrogen and accumulating P(3HB) within its cell, using fructose or CO_2_ as the sole carbon source. On the other hand, *R. eutropha* 1F2, a leucine analog-resistant mutant strain, produces 3HB-based copolymers containing 3HV and 3H4MV comonomer units via a branched amino acid synthesis/degradation pathway when expressing a broad substrate-specific PHA synthase [13,14,15]. However, the comonomer fraction remains low, often not exceeding 5 mol% [14,15]. Recent studies have highlighted the biosynthesis of a PHA copolymer containing a 3HV unit up to 64.9 mol% using CO_2_ as the sole carbon source by engineered *R. eutropha* H16 [16]. This achievement involved the heterologous expression of acetolactate synthase (AlsS), an enzyme facilitating the conversion of pyruvate to 2-acetolactate, thereby augmenting the supply of propionyl-CoA, the precursor of the 3HV monomer [16].

In the valine degradation pathway, isobutyryl-CoA undergoes conversion to 3HV-CoA through a series of enzymatic reactions involving intermediates such as 3H2MP-CoA and propionyl-CoA [17]. Consequently, it was hypothesized that the accumulation of isobutyryl-CoA within the cell may lead to an increase in the proportion of 3HV units present in the PHA copolymer. The enzymes 2-ketoacid decarboxylase (Kivd) and phenylacetaldehyde dehydrogenase (PadA) efficiently catalyze the production of isobutyric acid from 2-ketoisovalerate via isobutylaldehyde [17]. In *Escherichia coli* strains expressing Kivd and PadA, isobutyric acid has reached 11.7 g/L from glucose as the sole carbon source [17]. Therefore, it would be very interesting if PadA and Kivd could be used as monomer suppliers to synthesize PHA copolymers including 3HV and 3H4MV units.

**Figure 1:**
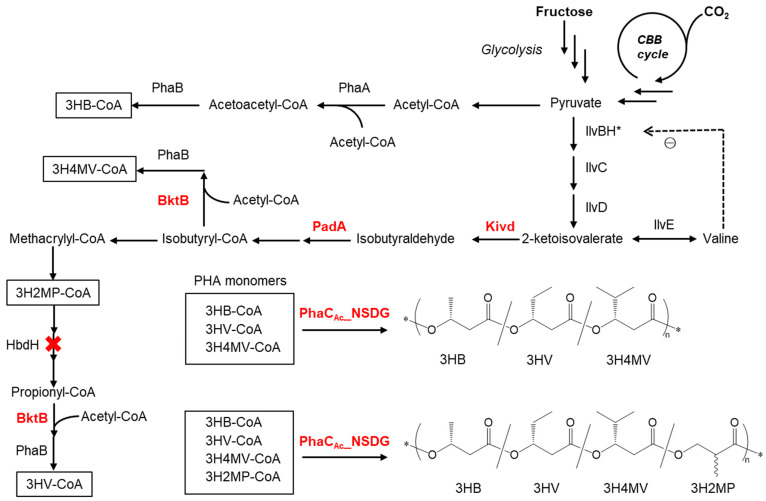
Presumed pathway for PHA copolymer biosynthesis from fructose and CO_2_ as a carbon source in the recombinant *R. eutropha* strain 1F2. The enzymes involved include Kivd (2-ketoacid decarboxylase from *L. lactis*), PadA (phenylacetaldehyde dehydrogenase from *E. coli*), BktB (3-ketothiolase), PhaA (3-ketothiolase), PhaB (NADPH-dependent acetoacetyl-CoA reductase), PhaC_Ac__NSDG (double mutant of PHA synthase from *A. caviae*), HbdH (3-hydroxyacyl-CoA dehydrogenase), IlvBH* (acetolactate synthase III and subunit protein, * denotes the introduction of A36T mutation in IlvH), IlvC (ketol-acid reductoisomerase), IlvD (dihydroxy acid dehydratase), and IlvE (branched-chain amino acid aminotransferase). ⊝, feedback inhibition.

Figure 1 illustrates the presumed metabolic pathways for the synthesis of PHA copolymers, starting from fructose and carbon dioxide if Kivd and PadA are expressed in *R. eutropha* 1F2. Part of the pyruvate is first converted to 3HB, and the remainder is converted to 2-ketoisovalerate. Kivd and PadA enhance the conversion of 2-ketoisovalerate to isobutyrate. Furthermore, isobutyrate is converted into isobutyryl-CoA and propionyl-CoA. Moreover, a broad substrate-specific 3-ketothiolase (BtkB) enhances the flux of acetopropionyl-CoA and acetoisobutyryl-CoA to form 3HV-CoA and 3H4MV-CoA, respectively. Finally, P(3HB-*co*-3HV-*co*-3H4MV) is polymerized by PHA synthase (PhaC). Since 3H2MP-CoA is considered to be an intermediate in the presumed 3HV biosynthetic pathway, a new PHA copolymer containing 3H2MP [13], an α-methylated monomer unit, can be incorporated into PHA copolymers. By deleting the 3-hydroxyacyl-CoA dehydrogenase gene (*hbdH*), whose gene product catalyzes the oxidation of 3-hydroxy-2-methylpropionate to methylmalonate semialdehyde in the valine degradation pathway [18], the synthesis of 3HV monomers can be inhibited, but a 3H2MP unit can be provided.

In this study, we aimed to synthesize a PHA copolymer comprising 3HV, 3H4MV, and 3H2MP units using fructose or CO_2_ as the sole carbon source by engineering *R. eutropha* 1F2 harboring a broad substrate-specific PHA synthase gene derived from *Aeromonas caviae* (*phaC_Ac__NSDG*). This gene product PhaC_Ac__NSDG has double mutants: Asparagine at position 149 is replaced by serine (N149S), and aspartic acid at position 171 is replaced by glycine (D171G) [19]. The double mutation in PhaC_Ac_ facilitates the polymerization of PHA with higher proportions of comonomer units. The engineered 1F2 strain expresses enzymes involved in monomer supply, including the overexpression of *bktB*, and the heterologous expression of *kivd* and *padA* leads to efficient incorporation of the targeted monomers into PHA. Furthermore, *hbdH* was deleted from the genome of strain 1F2 to enhance the supply of a 3H2MP unit instead of a 3HV unit. The biosynthesized PHAs were characterized in terms of chemical structures, chiral configurations, and molecular weights.

## 2. Materials and Methods

### 2.1. Bacterial Strains and Plasmids

*R. eutropha* 1F2, a mutant resistant to branched-chain amino acid analogs [13,14], and *R. eutropha* 1F2Δ*hbdH* (a strain deficient in 3-hydroxyacyl-CoA dehydrogenase) were utilized as host strains for PHA biosynthesis. To construct the *hbdH*-deleted strain, we conducted homologous recombination between the *hbdH* gene of *R. eutropha* 1F2 and the plasmid pK18-d-hbdH. Initially, the plasmid pK18-d-hbdH was introduced into *E. coli* S17-1 [20], known for its proficient plasmid transformation ability, using the conjugation method. The resulting transformant was cultured in Lysogeny broth (LB) medium (5 g/L yeast extract, 10 g/L peptone, and 10 g/L NaCl) supplemented with 50 mg/L kanamycin and incubated overnight at 37 °C. Simultaneously, *R. eutropha* 1F2 was cultured in the nutrient-rich (NR) medium (2 g/L yeast extract, 10 g/L Bacto tryptone, and 10 g/L bonito extract) and incubated overnight at 30 °C.

Subsequently, 2 mL of each culture medium was harvested and centrifuged at 10,000× *g* for 2 min to obtain the bacterial pellets. The pellets were washed three times with 1 mL NR medium to eliminate the residual antibiotics. The washed bacterial pellets were then resuspended in 50 μL of NR medium to prepare a mixed bacterial suspension. Next, 100 μL of the bacterial suspension was dropped onto NR agar plate and incubated for 24 h at 30 °C to facilitate conjugative transfer.

Following the incubation, the cultured bacteria were suspended in saline solution and inoculated onto Simmons citrate agar plates (2 g/L trisodium citrate dihydrate, 5 g/L NaCl, 1 g/L KH_2_PO_4_, 1 g/L NH_4_H_2_PO_4_, 0.2 g/L MgSO_4_·7H_2_O, and 20 g/L agar) supplemented with 200 mg/L kanamycin and 10 mg/L gentamicin. The plates were then incubated for 2 days at 30 °C to allow for colony formation. The resulting colonies were inoculated into NR medium (2 mL) containing 200 mg/L kanamycin and incubated overnight at 30 °C.

The cultured medium was suspended in saline solution to prepare a dilution, which was then inoculated into the mineral salt-supplemented with yeast extract (MSY) medium containing 150 g/L sucrose and incubated for 2 days at 30 °C. The high concentration of sucrose was used as a counter-selection marker in the second recombination event. The MSY medium is comprised of 9 g/L Na_2_HPO_4_·12H_2_O, 1.5 g/L KH_2_PO_4_, 0.5 g/L NH_4_Cl, 0.2 g/L MgSO_4_·7H_2_O, 1 mL of trace element solution, and 1 g/L yeast extract. The trace element solution contained 0.218 g/L CoCl_2_·6H_2_O, 20.5 g/L FeCl_3_·6H_2_O, 7.8 g/L CaCl_2_, 0.118 g/L NiCl_2_·6H_2_O, 0.105 g/L CrCl_3_·6H_2_O, and 0.156 g/L CuSO_4_·5H_2_O in 0.1 N HCl.

The resulting colonies underwent colony PCR using the following primers: forward primer: 5′-TCGACACCTACACGCTGCAGCAGCC-3′; reverse primer: 5′-TCCATCAGCGCCACCAGCGGCTTGG-3′ to confirm the construction of *R. eutropha* 1F2Δ*hbdH* (deletion of *hbdH* from the genome).

The plasmid pJRD215_P_Ac__*phaC_Ac_*_*NSDG*, containing a *phaC_Ac__NSDG* and *pha* promoter from *A. caviae*, was utilized for PHA biosynthesis, with these integrated at multiple cloning sites of pJRD215 [21]. The plasmid pBBR1MCS-3_P_tac_-*bktB* or pBBR1MCS-3_P_tac_-*bktB*-*kivd*-*padA* was employed to enhance the supply of comonomer units. The plasmid carries the *bktB* gene or *bktB*-*kivd-padA* genes inserted downstream of the *tac* promoter at the multi-cloning site of pBBR1MCS-3 [22]. The *kivd* (GenBank accession number: ADA65057) is a 2-ketoacid decarboxylase gene derived from *Lactococcus lactis* [23]. The *padA* (NP_415903) is a phenylacetaldehyde dehydrogenase gene derived from *E. coli* [17]. The *bktB* (H16_A1445) is a broad substrate-specific 3-ketothiolase gene derived from *R. eutropha* H16 [24].

### 2.2. Plasmids Construction

The suicide plasmid system of pK18-d-hbdH was constructed to delete the *hbdH* (H16_B1190) gene from the genome of *R. eutropha* 1F2. Genomic DNA from *R. eutropha* H16 was extracted using a Wizard Genomic DNA Purification Kit (Promega, Madison, WI, USA). The extracted genomic DNA was used as a template for PCR amplification using primers A to D (forward primer A: 5′-TTCGAATTCCCTTGGGGCCGGCGCCGCGCAGCAC-3′, reverse primer B: 5′-AGGAGACCCCATGATCGAGTTCGCCCTGCACGGCCACGTCG-3′, forward primer C: 5′-ACTCGATCATGGGGTCTCCTTCCGTGTCGTTCTTGAATGC -3′, and reverse primer D: 5′-GACGCATGCGACCCGCTGTCCACCGTGGAGCTGG-3′). This yielded 1 kb DNA fragments upstream and downstream of *hbdH.* Subsequently, overlap PCR was performed with primers A and D, using the obtained DNA fragments as templates. The 2 kb DNA fragment obtained was digested using the restriction enzymes *Sph* I and *Eco*R I, then inserted into the *Sph* I and *EcoR* I sites of pK18mobsacB [25], resulting in the construction of the plasmid pK18-d-hbdH (Figure S1).

To construct the plasmid pBBR1MC3_P_tac__*bktB-kivd-padA*, *kivd* derived from *L. lactis* [23] and *padA* derived from *E. coli* K-12 strain [17] were chemically synthesized (Table S1). The nucleotide sequence of the genes is shown in the Supplementary Information. Codons were optimized for *E. coli* expression, and chemical synthesis products were obtained from Eurofins Genomics Co., Ltd. Additionally, *bktB* (H16_A1445) was derived from *R. eutropha* H16 [24]. Initially, using *kivd* and *padA* as templates, PCR amplification was conducted with primers 1 to 4 (forward primer 1: 5′-TCTGAGGTTAGCCTTGGTACCGAAGGAGATATACA-3′, reverse primer 2: 5′-TGTATATCTCCTTCCTCGAGTCAGGATTTGTTCT-3′, forward primer 3: 5′-AGAACAAATCCTGACTCGAGGAAGGAGATATACA-3′, and reverse primer 4: 5′-AAAGGGAACAAAAGCTGGGTACCTCAATAGCGTAC-3′). The resulting PCR product served as a DNA template. For overlapping PCR, forward primer 1 and reverse primer 4 were utilized to generate the DNA fragment of *kivd-padA*, which measured 3.2 kb. Subsequently, the DNA fragment of *kivd-padA* was digested with the restriction enzymes *Kpn* I and inserted into the *Kpn* I site of pBBR1”C1ABP_tac_BktB [14], thereby constructing the plasmid pBBR1”C1_Ps_AB_Re_P_tac_-*bktB-kivd-padA* (14.5 kb).

Next, using pBBR1”C1_Ps_AB_Re_P_tac_-*bktB-kivd-padA* as the DNA template, PCR amplification was performed with primers 5 to 8 (forward primer 5: 5′-AAAGGGAACAAAAGCTGGGTACCTCAATAGCGTAC-3′, reverse primer 6: 5′-TTCGAGCGTATCTGAGGTTAGCCTTGAAGGAGATA-3′, forward primer 7: 5′-ATGTATATCTCCTTCAAGGCTAACCTCAGATACGC-3′, and reverse primer 8: 5′-GGCCGCTCTAGAACTAGTGGATCCCCCGGGCTG-3′). This process yielded a *tac* promoter (P_tac_) and *bktB*, *kivd*, and *padA* gene fragments.

Finally, the broad-host vector pBBR1MCS-3 (5.2 kbp) [22] was digested with the *Kpn* I and *Spe* I. The P_tac_, *bktB*, *kivd*, and *padA* gene fragments were integrated into pBBR1MCS-3 utilizing the In-Fusion HD Cloning Kit (Takara Bio Co., Ltd., Ohtsu, Japan), yielding the plasmid pBBRMCS-3_P_tac_-*bktB-kivd-padA* (9.8 kbp) (Figure S2).

To construct the plasmid pBBRMCS-3_P_tac_-*bktB*, the plasmid pBBRMCS-3_P_tac_-*bktB-kivd-padA* underwent digestion with the *Kpn* I, followed by self-ligation.

To construct the plasmid pJRD215_P_Ac__*phaC_Ac_*_*NSDG*, pBBREE”P_Ac_NSDG vector (7.3 kb) [26] and pJRD215 vector [21] were digested with the *Eco*R I and *Bam*H I. Additionally, the *pha* promoter of *A. caviae* FA440 and the *phaC_Ac_*_*NSDG* gene were inserted into the *Eco*R I and *Bam*H I sites of the pJRD215 vector, resulting in plasmid pJRD215_P_Ac_-*phaC_Ac__NSDG* (12.1 kbp) for PHA synthesis (Figure S3).

Details of plasmid construction are also described in the Supplementary Information.

### 2.3. Biosynthesis of PHA Copolymers from Fructose or CO_2_

The recombinant cells were grown on NR agar plates with antibiotics overnight at 30 °C. The cells were inoculated in a 10 mL test tube with 2 mL of NR medium with antibiotics and incubated for 15–20 h for pre-cultivation. For heterotrophic cultivation, 1 vol% of the pre-cultivation was inoculated into a 500 mL shake flask with 100 mL of mineral salt (MS) medium (9 g/L Na_2_HPO_4_·12H_2_O, 1.5 g/L KH_2_PO_4_, 0.5 g/L NH_4_Cl, 0.2 g/L MgSO_4_·7H_2_O, and 1 mL trace element solution) with 10 g/L fructose serving as the sole carbon source. Antibiotics (100 mg/L kanamycin and 10 mg/L tetracycline) were added to the medium to maintain the plasmids. The flask was incubated at 130 rpm for 72 h at 30 °C. For autotrophic cultivation, 1 vol% of the pre-cultivation was inoculated into a 250 mL jar fermenter (Bio Jr8, Able Corp., Tokyo, Japan). The fermenter contained 100 mL of MS medium, and CO_2_ was used as the sole carbon source. The cultivation medium was supplemented with 0.01% (*w*/*v*) of antifoam 204 (Sigma Aldrich, St. Louis, MO, USA) to avoid foam formation during the cultivation. Antibiotics (100 mg/L kanamycin and 10 mg/L tetracycline) were added to the medium to maintain plasmid expression. Autotrophic cultivation was carried out with a continuous gas flow system under non-combustible conditions [27]. The gas mixture for autotrophic growth consisted of a gas cylinder (5.7% H_2_, 20.1% CO_2_, and 74.2% N_2_) and an air compressor (atmosphere) at 2: 1 flow ratio equipped with mass flow controllers (SEC-E40, CU-2130, S48 32, and MT-51; Horiba, Tokyo, Japan), resulting in a gas mixture (H_2_: O_2_: CO_2_: N_2_: = 3.8: 7.3: 13.0: 75.9 vol%). The gas mixture was delivered to a 250 mL jar fermenter at 5 mL/min of gas flow rate. As fermentation proceeded, 1 mL of the cultivation broth was sampled at different time intervals to measure the turbidity at a wavelength of 600 nm (OD_600_) using a UV–vis spectrophotometer.

### 2.4. Analysis of PHA

#### 2.4.1. PHA Content Analysis by Gas Chromatography (GC)

Intracellular PHA content was determined by gas chromatography (GC) using a GC-2014s (Shimadzu, Kyoto, Japan) equipped with an inert cap1 (GL Sciences, Tokyo, Japan). Before GC analysis, the PHA sample underwent methanolysis using sulfuric acid and methanol to break it down into monomeric units. For sample preparation, approximately 15–20 mg of dry cells were weighed into a glass tube with a screw cap. Then, 2 mL of sulfuric acid methanol (sulfuric acid:methanol = 15:85) and 2 mL of chloroform were added. The tube was heated at 100 °C for 140 min and shaken every 30 min during the reaction time. The reaction mixture was then cooled to room temperature. Subsequently, 1 mL of water was added to separate the organic layer (lower layer) from the aqueous layer (upper layer). The lower layer was aspirated using a glass pipette and filtered through a 0.45 μm regenerated cellulose membrane. Then, 500 μL of the filtered liquid and 500 μL the internal standard solution (chloroform solution with 0.1% (*v*/*v*) octanoic acid methyl ester (Kanto Chemical Co., Inc., Tokyo, Japan)) were put in a glass vial for GC analysis. One μL of the GC sample was injected into the GC device. The heating program of the GC device was kept at 90 °C for 2 min, heated to 110 °C at 5 °C/min, heated to 280 °C at 20 °C/min, and kept for 5 min. For data analysis, intracellular PHA amounts and composition ratios were calculated from the peak areas considering each detection sensitivity.

#### 2.4.2. Structure Analysis by Gas Chromatography–Mass Spectrometry (GC-MS)

The PHA compositions of the samples were analyzed using gas chromatography–mass spectrometry (GC-MS; QP2010, Shimadzu). A Beta-DEX 120 column (fused silica capillary column; length, 30 m; inner diameter, 0.25 mm; film thickness, 0.25 μm; Supelco, Sigma Aldrich) was used for the chiral monomer separation. The temperature program was kept at a constant oven temperature of 85 °C for 15 min, then raised to 280 °C at 20 °C/min, and finally maintained at 280 °C for 5 min to eliminate chemical residues in the column. Methyl (*R*)-3H2MP and methyl (*S*)-3H2MP (Tokyo Kasei Kogyo, Co., Tokyo, Japan) were the standards, while approximately 3 mg of PHA samples were methanolyzed in 15% (*v*/*v*) sulfuric acid/methanol at 100 °C for 140 min. The enantiomeric molar ratio was calculated from the peak area at *m*/*z* 88 for each enantiomer in the MS spectrum.

#### 2.4.3. Molecular Weight Analysis by Gel Permeation Chromatography (GPC)

Intracellular PHA was extracted using sodium dodecyl sulfate (SDS) and sonication treatment [28]. Approximately 50–100 mg of lyophilized cells were suspended in a 4 wt.% SDS solution. The samples were then ultrasonicated at 12 W for 10 min. In addition, the tubes containing samples were immersed in water to prevent high-temperature sonication. Subsequently, the pellets were centrifuged at 10,000× *g* for 10 min and rinsed at least three times with distilled water. Finally, the polymer pellets were obtained by freeze drying for 24 h. The molecular weight of PHA was measured using GPC. The sample was prepared by dissolving the polymer pellets in chloroform at a concentration of 1 mg/mL and then filtering through a 0.45 μm PVDF filter membrane. GPC analysis was performed using a Shimadzu Nexera 40 GPC system and Shodex RI-504 refractive index detector (Showa Denko, Tokyo, Japan) with two joint columns of Shodex GPC KF-406LHQ. Chloroform was the eluent at 0.3 mL/min. Calibration curves were constructed using polystyrene with a low polydispersity.

#### 2.4.4. Structure Analysis by Nuclear Magnetic Resonance (NMR)

The chemical structure of the biosynthesized PHA was verified by ^1^H NMR analysis using a 500 MHz AVANCE III spectrometer (Bruker BioSpin, Rheinstetten, Germany). Approximately 10 mg of the polymer sample was dissolved in 1 mL of *d*-chloroform and filtered through a 0.45 μm PVDF filter membrane. Subsequently, 700 μL of the sample was injected into an NMR tube.

## 3. Results

### 3.1. Biosynthesis of PHA by Recombinant R. eutropha 1F2 from Fructose

First, recombinant *R. eutropha* 1F2 harboring two plasmids, pJRD215_P_Ac_-*phaC_Ac__NSDG* and the pBBR1MCS-3-derived plasmid, was used for the biosynthesis of PHA copolymers. The culture results obtained using fructose as the sole carbon source are shown in Table 1. The recombinant *R. eutropha* 1F2 expressing PhaC_Ac__NSDG accumulated 54.4 wt.% of PHA in the cells. The biosynthesized PHA copolymer was composed of 98.5 mol% 3HB, 1.2 mol% 3HV, and 0.3 mol% 3H4MV (Entry 1). Notably, in the *bktB*-dosed strain (Entry 2), PHA content increased to 66.9 wt.%, and the 3HV fraction has tripled (3.6 mol%) compared with Entry 1. Additionally, upon the introduction of the *kivd* and *padA* genes (Entry 3), the 3HV fraction increased significantly to 13.9 mol%, resulting in the total comonomers reaching 14.4 mol%. The PHA content in the cells was the same as that in Entry 1 but decreased from Entry 2.

### 3.2. NMR Analysis of Biosynthesized PHA

**Figure 2:**
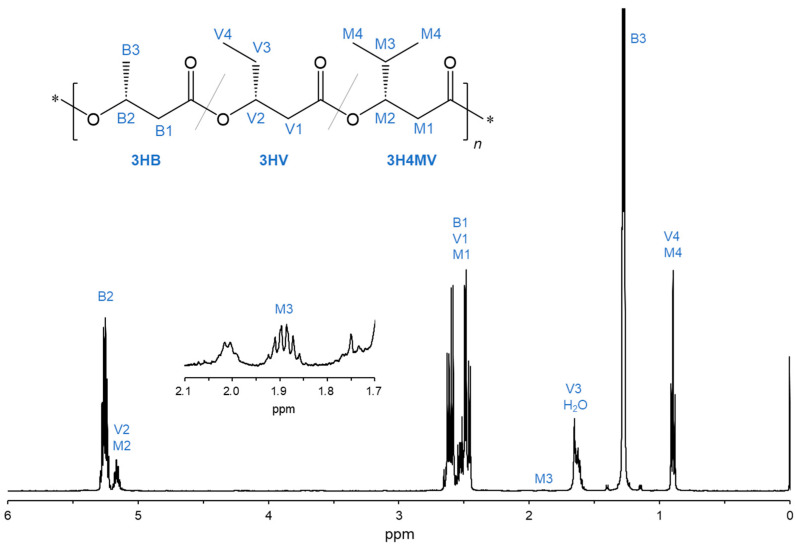
^1^H NMR spectra (500 MHz) of the biosynthesized PHA copolymer by cultivating the recombinant *R. eutropha* 1F2 strain expressing *bktB*, *kivd*, *padA*, and *phaC_Ac__NSDG* genes (Entry 3).

The chemical structure of the PHA copolymer biosynthesized by *R. eutropha* 1F2 expressing BktB, Kivd, and PadA (Entry 3) was analyzed using NMR. Figure 2 shows the 500 MHz ^1^H NMR spectrum with the assigned proton resonance. The signals of the methyl protons (-CH_3_) of 3HV and 3H4MV (V4 and M4, 0.90 ppm) and the methine proton (-CH<) of 3H4MV (M3, 1.89 ppm) were detected in ^1^H NMR spectrum. This indicates that 3HV and 3H4MV units were included in the biosynthesized PHA copolymer (Entry 3). The monomer composition was calculated based on the peak intensity of signals B2, M3, and V4 + M4, resulting in the copolymer composition of P(3HB-*co*-15.7 mol% 3HV-*co*-0.52 mol% 3H4MV).

### 3.3. Biosynthesis of 3H2MP-Containing PHA Copolymer Using the hbdH-Deficient Strain

3-Hydroxy-2-methylpropionyl-CoA (3H2MP-CoA), an intermediate in the valine degradation pathway, acts as a precursor to α-methylated PHA, representing a novel bio-based material. α-Methylated PHA exhibits favorable material properties such as high thermal stability and fast crystallization [9,10,11,12]. To incorporate the 3H2MP unit into the 3HB-based copolymer, the 3-hydroxyacyl-CoA dehydrogenase gene (*hbdH*: H16_B1190), which participates in the valine degradation pathway, was removed from the genome, leading to the development of a *hbdH*-deficient strain. A PHA copolymer with 0.9 mol% of 3H2MP unit was obtained by cultivating a *hbdH*-deficient strain expressing PhaC_Ac__NSDG using 10 g/L fructose (Table 1). In strains expressing Kivd and PadA, the 3H2MP and 3H4MV fractions were increased by 1.9-fold and 6.5-fold, respectively, reaching 1.7 mol% for 3H2MP and 1.3 mol% for 3H4MV (Entry 6). Contrastingly, the 3HV fraction and PHA accumulation decreased to 2.2 mol% and 7.8 wt.%, respectively, compared to the *hbdH* non-deletion strain (Entry 3).

### 3.4. Structural Analysis of PHA Biosynthesized by hbdH-Deficient Strains

**Figure 3:**
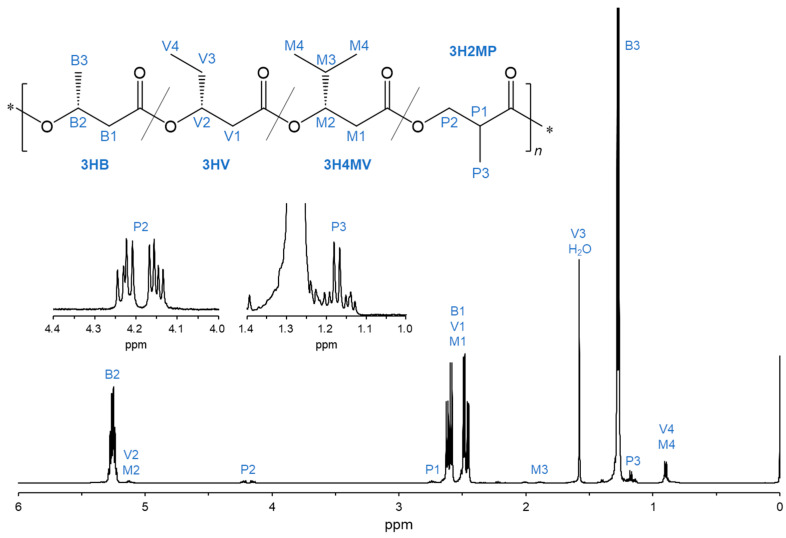
^1^H NMR spectra (500 MHz) of the biosynthesized PHA copolymer by cultivating the recombinant *R. eutropha* 1F2ΔhbdH expressing *bktB*, *kivd*, *padA*, and *phaC_Ac__NSDG* genes (Entry 6).

The chemical structures of the PHA copolymers incorporating the 3H2MP unit were investigated through ^1^H NMR analysis. The signals corresponding to the methyl proton (-CH_3_), the methine (-CH<), and the methylene (-CH_2_-) of the 3H2MP monomer were detected at 1.16–1.18 ppm (P3), 2.71–2.76 ppm (P1), and 4.13–4.25 ppm (P2) in the ^1^H NMR spectrum, respectively (Figure 3). This result indicated that the 3H2MP monomer was incorporated into the PHA copolymer.

**Figure 4:**
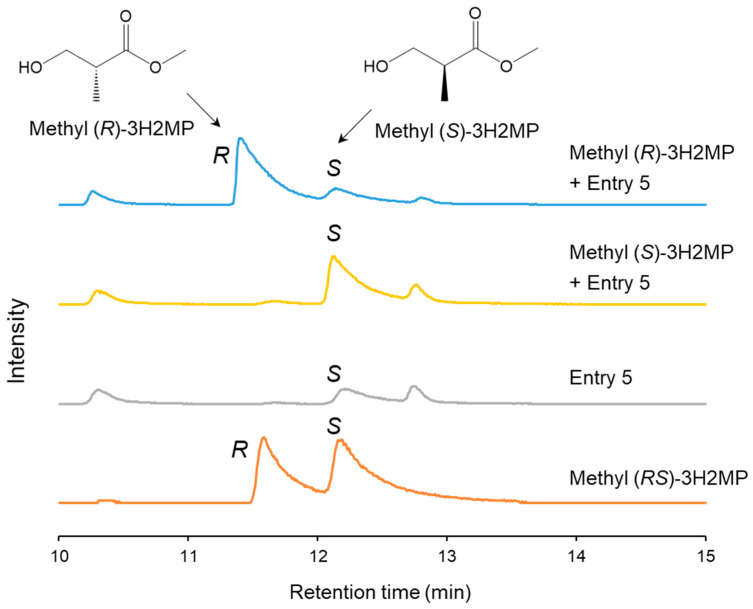
The *m*/*z* 88 ion chromatograms of biosynthesized PHA and methyl (*S*, *R*, and *RS*)-3H2MP by GC-MS equipped with a chiral separation column. The biosynthesized PHA was extracted from dry cells of the recombinant *R. eutropha* 1F2*ΔhbdH* harboring pJRD215_P_Ac_-*phaC*_*NSDG* and pBBR1MCS-3_P_tac_-*bktB* (Entry 5).

The biosynthesized α-methylated monomers possess chiral characteristics due to the chiral center on their α-carbon. The methyl-esterified PHA monomers underwent separation using a gas chromatograph–mass spectrometer equipped with a chiral separation column. Methyl (*RS*)-3H2MP, methyl (*S*)-3H2MP, and methyl (*R*)-3H2MP were used as the standards. The GC-MS ion spectra of *m*/*z* 88, which is the characteristic fragment of methyl 3H2MP, exhibited a distinct separation of the two enantiomers of methyl (*RS*)-3H2MP. The retention times were 11.8 min for methyl (*R*)-3H2MP and 12.4 min for methyl (*S*)-3H2MP, respectively, as shown in Figure 4. Furthermore, by conducting analyses with the addition of either methyl (*S*)-3H2MP or methyl (*R*)-3H2MP standards to Entry 5, we determined that the 3H2MP contained within the PHA biosynthesized by the *hbdH*-deficient strain consisted of the 2*S* enantiomer.

### 3.5. Molecular Weight of PHA Biosynthesized from Fructose

**Figure 5:**
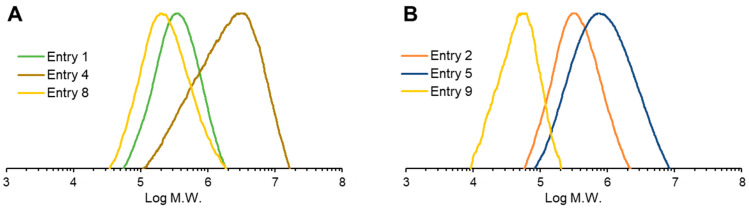
The molecular weight distribution of the biosynthesized PHA. (A) Non-*bktB*-dosed strains: strain 1F2 on fructose (Entry 1), strain 1F2 *ΔhbdH* on fructose (Entry 4), and strain 1F2 on CO_2_ (Entry 8). (B) *bktB*-dosed strains: strain 1F2 on fructose (Entry 2), strain 1F2 *ΔhbdH* on fructose (Entry 5), and strain 1F2 *ΔhbdH* on CO_2_ (Entry 9).

The weight-average molecular weight (*M_w_*) of the PHA copolymers was determined by GPC analysis. As indicated in Table 1, in the case of the recombinant strain without *hbdH* deletion, *M_w_* ranged from 3.40 × 10^5^ to 4.89 × 10^5^, while the polydispersity index (PDI) ranged from 1.86 to 1.95. Surprisingly, the molecular weight of the PHA biosynthesized by *hbdH*-deficient strains was substantially increased up to 32.50 × 10^5^ for *M_w_*. Moreover, the PDI of the PHA copolymers was higher than that of the strain without the *hbdH* gene deletion (Figure 5).

### 3.6. Biosynthesis of PHA from CO_2_

*R. eutropha* is a hydrogen-oxidizing chemoautotrophic bacterium capable of using CO_2_ as its sole carbon source by utilizing the supplied hydrogen and oxygen. Under autotrophic conditions, *R. eutropha* 1F2 expressing BktB, Kivd, and PadA was cultured alongside two *hbdH*-deficient strains, which exhibited significant PHA copolymers accumulation from fructose. The autotrophic culture results are shown in Table 2. Consequently, the PHA copolymer accumulated at 28 wt.% in the cells, containing 6.4 mol% 3HV and 0.9 mol% 3H4MV monomers (Entry 7). Compared with the results of the heterotrophic culture (Table 1), both PHA content and 3HV fraction were halved. In contrast, the *hbdH*-deficient strain produced a 3H2MP-containing copolymer, P(3HB-*co*-1.2 mol% 3H4MV-*co*-1.4 mol% 3H2MP) (Entry 9), even when cultivated with carbon dioxide as the carbon source. Due to the deletion of *hbdH*, the 3HV unit was not detected in the PHA copolymer (Entries 8 and 9). The PHA content in the *hbdH*-deficient strains was 49.0–49.7 wt.%, which is a lower level than the heterotrophic culture conditions (58.2–60.5 wt.%).

The molecular weights and disparities in PHA biosynthesis from CO_2_ are shown in Table 2 and Figure 5. The *M_w_*s of PHA was (0.59–3.56) × 10^5^, which is not as high as that of the heterotrophic culture.

## 4. Discussion

A multitude of PHA copolymers have been synthesized from biomass resources utilizing *R. eutropha* in conjunction with precursors. However, the addition of precursors is costly and often inhibits cell growth and PHA biosynthesis [13,14,15]. Therefore, it is necessary to develop a technology for biosynthesizing PHA copolymers from a sole carbon source without using precursors. *R. eutropha* 1F2 was constructed from *R. eutropha* PHB^−^4 using random chemical mutagenesis [13]. This mutant strain harbors a mutation in the *ilvH* gene (acetolactate synthase III small subunit gene) at amino acid position 36 (A36T), attributing tolerance to the feedback inhibition of branched amino acids, resulting in the 1F2 strain exhibiting the distinct characteristic of suppressed feedback inhibition against branched amino acids [13,14,15]. Therefore, this mutant strain plays a pivotal role in facilitating the biosynthesis of comonomer units such as 3HV and 3H4MV via valine degradation.

In previous studies [13,14,15], although 3HB-based copolymers containing 3HV and 3H4MV could be biosynthesized using fructose or CO_2_ as the sole carbon source, the synthesis resulted in only a small quantity of comonomer units. This was due to the insufficient supply of the comonomer within the cells. This study illustrates that the insertion of *bktB, kivd*, and *padA* genes augmented the integration of comonomer units into PHA copolymers (Table 1). In particular, the heterologous expression of Kivd and PadA contributed to a notable increase in the proportion of 3HV monomers. This increase was attributed to Kivd, facilitating the conversion of 2-ketoisovalerate to isobutyraldehyde, and subsequently, PadA increased the conversion flux to isobutyrate, resulting in elevated 3HV-CoA levels in the cells (Figure 1). Kivd and PadA have been studied for their high-efficiency production of isobutyrate and isobutanol, achieving 90 g/L of isobutyrate [17,29] and 275 g/L of isobutanol [30] in recombinant *E. coli* (in vivo) and cell-free (in vitro) systems, respectively. Moreover, the introduction of amino acid mutations (V461I or S286T) in Kivd has been reported to enhance decarboxylase activity [31]. This study pioneers a novel approach that will expedite future PHA field research by applying Kivd and PadA to PHA production. Moreover, BktB facilitates the condensation reaction between propionyl-CoA and acetyl-CoA, resulting in the production of 3-oxovaleryl-CoA. Subsequently, this compound is reduced to 3HV-CoA by NADPH-dependent acetoacetyl-CoA reductase (PhaB). Consequently, the overexpression of BktB enhanced the proportion of the 3HV fraction (Table 1), which is consistent with a previous study [14].

HbdH plays a crucial role in the valine degradation pathway by catalyzing the oxidation of 3-hydroxy-2-methylpropionic acid (3H2MP) to methylmalonic semialdehyde, which is subsequently converted to 3HV-CoA via several enzymatic reactions [18]. The 3H2MP-CoA generated through the valine degradation pathway serves as a precursor to the α-methylated PHA monomer. Consequently, the *hbdH* deletion resulted in the biosynthesis of α-methylated PHA containing 3H2MP units while effectively reducing the supply of 3HV monomer (Table 1). In addition, the accumulation of isobutyraldehyde and/or isobutyrate, which are toxic to organisms, in cells by Kivd-PadA expression may inhibit cell growth and PHA synthesis [32,33]. Hence, PHA content decreased in the *hbdH*-deficient strain expressing Kivd-PadA (Table 1).

The molecular weight of PHA is a critical determinant of the material characteristics of the resulting polymer. In general, an elevated molecular weight is associated with enhanced material properties. Notably, ultrahigh-molecular-weight P(3HB), characterized by molecular weights reaching three million, shows outstanding mechanical properties and high usefulness [34,35]. In the PHA production employing the engineered *R. eutropha* 1F2 strain, the *M_w_* of the resulting PHA ranged from 3.40 × 10^5^ to 4.89 × 10^5^, aligning with the typical molecular weight observed in conventional PHA biosynthesis [13,15]. In contrast, in the *hbdh*-deficient strains, the *M_w_* increased by an order of magnitude, reaching up to 32.50 × 10^5^. In PHA biosynthesis, the presence of certain hydroxyl compounds, such as ethanol and propanol [36], within cells can induce a chain transfer (CT) reaction that terminates PHA polymerization [37,38,39]. Consequently, PHAs with lower molecular weights were produced [39]. Typically, propionyl-CoA, originating from the valine degradation pathway, undergoes metabolism via the methylcitrate cycle and/or tricarboxylic acid cycle, resulting in the synthesis of a wide range of chemical compounds [18]. Therefore, in *hbdh*-deficient strains, the suppression of valine degradation may suppress the production of certain CT agents, consequently increasing the molecular weight of the synthesized PHA (Table 1).

Furthermore, using CO_2_ as the sole carbon source, autotrophic cultivation yielded poorer cell growth and PHA accumulation compared to heterotrophic cultivation with fructose as the sole carbon source. This difference arises from *R. eutropha*’s preference for utilizing fructose over CO_2_ in our culture conditions. Utilizing CO_2_ requires a more intricate and energy-intensive carbon fixation process through the Calvin–Benson–Bassham (CBB) cycle, which involves the pivotal enzyme ribulose-1,5-bisphosphate carboxylase/oxygenase (RuBisCO) [40]. Thus, enhancing the CO_2_ uptake by *R. eutropha* and its utilization efficiency is a primary challenge in refining autotrophic cultivation. Interestingly, various strategies have been proposed to address this challenge, including the enhancement of CO_2_ uptake into cells by the gene dosage of carbonic anhydrase [41,42,43,44], engineering the CBB cycle and hydrogen utilization pathway of *R. eutropha* [45], engineering a rhodopsin-based photoelectrosynthesis system in *R. eutropha* [42], and coupling chemical processes for CO_2_ reduction with biological processes for PHA synthesis [46,47]. These studies provide valuable insights and promising avenues for further improvement in enhancing PHA accumulation, particularly in copolymer synthesis from CO_2_ using hydrogen-oxidizing bacteria.

## 5. Conclusions

This study demonstrated that the overexpression of the homologous broad substrate-specific 3-ketothiolase gene (*bktB*), heterologous expression of the 2-ketoacid decarboxylase gene (*kivd*), and the phenylacetaldehyde dehydrogenase gene (*padA*) contribute to enhancing the proportion of comonomer units. Additionally, deletion of the 3-hydroxyacyl-CoA dehydrogenase gene (*hbdH*) from the genome resulted in *R. eutropha* 1F2 biosynthesizing the 3H2MP-containing copolymer without the addition of any external precursors. However, excessive expression of these genes may cause toxic substances such as isobutyraldehyde to accumulate in the cell. Hence, future directions for this study could involve controlling the expression level of *bktB-kivd-padA* and identifying a suitable enzyme to transform 3H2MP-CoA.

## Figures and Tables

**Table 1:** Biosynthesis of PHA copolymers from fructose by recombinant *R. eutropha* 1F2 harboring *phaC_Ac__NSDG*.

Entry	Genome	Plasmid-Based Expression	Dry Cell wt. (g/L)	PHA Content (wt.%)	PHA (g/L)	PHA Composition (mol%) ^a^				*M_w_* (×10^5^) ^b^	PDI ^b^
						3HB	3HV	3H4MV	3H2MP		
1	-	*-* ^c^	1.59 ± 0.04	54.4 ± 1.3	0.86 ± 0.04	98.5	1.2	0.3	0	4.62	1.86
2	-	*bktB*	1.93 ± 0.05	66.9 ± 2.1	1.29 ± 0.05	96.0	3.6	0.4	0	4.89	1.92
3	-	*bktB*, *kivd*, *padA*	1.67 ± 0.03	55.9 ± 1.8	0.93 ± 0.03	85.6	13.9	0.5	0	3.40	1.95
4	*ΔhbdH*	- ^c^	2.10 ± 0.01	58.2 ± 2.0	1.22 ± 0.04	97.8	1.1	0.2	0.9	32.50	3.64
5	*ΔhbdH*	*bktB*	1.97 ± 0.07	60.5 ± 2.0	1.19 ± 0.07	97.6	0.9	0.4	1.1	14.80	2.89
6	*ΔhbdH*	*bktB*, *kivd*, *padA*	0.97 ± 0.02	7.8 ± 0.6	0.08 ± 0.04	94.8	2.2	1.3	1.7	3.97	2.11

The cells were cultured in a 500 mL shake flask with 100 mL MS medium containing 10 g/L fructose at 30 °C for 72 h. All strains were cultivated in triplicate. ^a^ Determined by GC analysis. ^b^ Determined by GPC analysis. PDI, polydispersity index. ^c^ Empty plasmid (pBBR1MCS-3) was used as a control.

**Table 2:** Biosynthesis of PHA copolymers from CO_2_ by recombinant *R. eutropha* 1F2 harboring *phaC_Ac__NSDG*.

Entry	Genome	Plasmid-Based Expression	Dry Cell wt. (g/L)	PHA Content (wt.%)	PHA (g/L)	PHA Composition (mol%) ^a^				*M_w_* (×10^5^) ^b^	PDI ^b^
						3HB	3HV	3H4MV	3H2MP		
7	-	*bktB*, *kivd*, *padA*	0.72	28.4	0.20	92.7	6.4	0.9	0	1.88	2.04
8	*ΔhbdH*	*-*	1.20	49.7	0.60	99.3	0	0.7	trace	3.56	2.24
9	*ΔhbdH*	*bktB*	1.14	49.0	0.55	97.4	0	1.2	1.4	0.59	1.64

The cells were cultivated in a 250 mL jar fermenter with 100 mL MS medium supplying a low-hydrogen-content gas mixture (3.8% H_2_, 7.3% O_2_, 13.0% CO_2_, and 75.9% N_2_) at 5 mL/min of gas flow rate, at 30 °C for 162 h [27]. ^a^ Determined by GC analysis. ^b^ Determined by GPC analysis. PDI, polydispersity index.

## Data Availability

All data can be found in the article.

## Supplementary Materials

The following supporting information can be downloaded at: https://www.mdpi.com/article/10.3390/bioengineering11050455/s1, Figure S1: Construction of the suicide plasmid of pK18-d-hbdH; Figure S2: Construction of pBBR1”C1_Ps_AB_Re_P_tac_-*bktB-kivd-padA* and pBBRMCS-3_P_tac_-*bktB-kivd-padA*; Figure S3: Construction of pJRD215_P_Ac__*phaC_Ac_*_*NSDG*; Table S1: Nucleotide sequence of the genes chemically synthesized in this study.
