# Supplementary material for: Polyhydroxyalkanoate Copolymer Production by Recombinant Ralstonia eutropha Strain 1F2 from Fructose or Carbon Dioxide as Sole Carbon Source
Source: Bioengineering (Basel). 2024 May 2;11(5):455. doi: 10.3390/bioengineering11050455 (PMC11117859; doi:10.3390/bioengineering11050455)
Supplement: Supplementary file 1 [file bioengineering-11-00455-s001.zip › bioengineering-2965757-supplementary.pdf]

**Supplementary information**

# Polyhydroxyalkanoate Copolymer Production by Recombinant *Ralstonia eutropha* Strain 1F2 from Fructose or Carbon Dioxide as Sole Carbon Source

**Chih-Ting Wang, Ramamoorthi M Sivashankari, Yuki Miyahara *, and Takeharu Tsuge ***

Department of Materials Science and Engineering, Tokyo Institute of Technology, 4259 Nagatsuta, Midori-ku, Yokohama 226-8502, Japan;
wang.c.aq@m.titech.ac.jp (C.-T.W.); sivashankari.m.aa@m.titech.ac.jp (R.M.S.)
* Correspondence: miyahara.y.aa@m.titech.ac.jp (Y.M.); tsuge.t.aa@m.titech.ac.jp (T.T.); Tel.: +81-45-924-5420 (T.T.)

**Table S1** Nucleotide sequence of the genes chemically synthesized in this study.

| *kivd* gene |
|---|
| ATGTATACGGTTGGCGATTACCTGTTAGATCGTCTGCATGAGTTAGGCATCG<br>AGGAAATTTTTGGAGTACCAGGTGACTACAATCTGCAGTTTCTGGATCAGA<br>TTATTTCGCGCAAAGACATGAAATGGGTGGGAAATGCGAACGAATTGAATG<br>CCTCATATATGGCCGACGGCTATGCACGGACCAAAAAAGCAGCCGCCTTTC<br>TGACGACATTTGGCGTCGGGGAATTGAGCGCCGTTAATGGCTTGGCGGGTA<br>GCTACGCGGAAAATCTTCCGGTTGTGGAGATTGTTGGTAGTCCTACCTCTA<br>AAGTCCAGAACGAGGGCAAATTCGTGCACCATACCCTTGCGGATGGCGATT<br>TCAAACACTTTATGAAAATGCATGAACCGGTTACCGCTGCGAGAACCCTGC<br>TGACGGCAGAAAACGCCACCGTCGAGATTGACCGTGTTTTGAGCGCTCTC<br>CTGAAAGAGCGCAAACCGGTGTACATCAACTTGCCAGTGGATGTCGCAGC<br>CGCAAAAGCGGAAAAACCCAGTTTACCTCTGAAGAAAGAAAATCCGACGT<br>CGAATACGAGCGATCAGGAGATCCTGAACAAAATTCAGGAAAGTCTGAAG<br>AATGCGAAAAAACCGATTGTCATAACAGGCCACGAAATTATTTCCTTTGGC<br>CTGGAAAACACCGTGACTCAGTTCATCTCGAAAACCAAATTGCCCATTACT<br>ACCCTTAACTTCGGGAAGTCTTCCGTGGACGAGACACTGCCGTCATTTTTA<br>GGGATCTACAATGGCAAACTGAGTGAACCGAATCTCAAAGAGTTCGTAGA<br>ATCTGCCGATTTCATCCTCATGCTGGGCGTAAAACTTACCGATAGCTCAACC<br>GGTGCGTTTACTCACCATCTTAACGAGAACAAGATGATTTCGCTGAATATCG |

ATGAAGGTAAGATATTCAACGAAAGCATCCAGAATTTCGACTTCGAAAGCC
TGATTTCCAGTCTGCTCGATTTGAGCGGTATTGAATACAAAGGCAAATATAT
TGACAAGAAACAGGAGGATTTTGTACCGAGCAACGCGTTACTGAGTCAAG
ATCGACTGTGGCAAGCTGTAGAAAATCTGACGCAAAGCAACGAAACGATA
GTTGCAGAACAGGGTACTTCGTTCTTTGGTGCCAGTTCTATTTTTCTCAAAC
CGAAATCCCACTTCATCGGTCAACCGCTGTGGGGTTCTATCGGGTATACCTT
TCCTGCGGCGCTAGGATCGCAAATTGCCGACAAAGAATCACGCCATCTGCT
GTTTATTGGCGATGGCTCTCTGCAACTGACTGTGCAGGAACTGGGCCTAGC
TATTCGCGAAAAGATCAATCCCATCTGCTTTATCATCAACAACGACGGTTAT
ACAGTTGAACGCGAGATCCATGGTCCAAACCAGTCCTATAACGACATTCCT
ATGTGGAACTATAGCAAATTACCGGAATCGTTCGGAGCTACCGAGGAACGT
GTGGTGAGCAAGATTGTGCGTACGGAAAACGAGTTTGTCTCAGTCATGAA
AGAAGCACAAGCAGATCCGAATCGTATGTATTGGATCGAACTGGTTCTTGC
GAAAGAAGATGCGCCAAAAGTGCTCAAGAAAATGGGGAAGTTATTTGCTG
AACAGAACAAATCCTGA

*padA* gene

ATGACCGAACCACATGTCGCGGTCTTGAGCCAGGTGCAACAGTTCCTCGAT
CGTCAACATGGGTTGTACATCGATGGTCGCCCAGGTCCTGCCCAGAGTGAG
AAACGTCTTGCTATCTTTGATCCAGCTACGGGTCAGGAAATTGCGTCGACC
GCGGATGCCAACGAAGCGGACGTGGACAACGCAGTTATGTCCGCTTGGCG
CGCTTTTGTGTCGCGCCGTTGGGCAGGCCGGCTTCCAGCGGAACGTGAAC
GCATTCTGTTGCGCTTTGCCGATTTGGTCGAACAGCACAGCGAAGAGCTG
GCACAGCTGGAGACACTCGAACAGGGCAAAAGCATCGCCATTTCGCGTGC
CTTTGAAGTTGGTTGCACGCTGAACTGGATGCGGTATACAGCCGGGCTCAC
CACCAAAATCGCGGGCAAAACGTTGGACCTGTCTATTCCCTTACCGCAAGG
TGCCCGCTATCAAGCCTGGACTCGCAAAGAACCGGTGGGTGTGGTTGCGG
GCATTGTCCCGTGGAATTTTCCCCTGATGATTGGTATGTGGAAAGTAATGCC
CGCTCTTGCAGCCGGTTGCAGCATTGTCATTAAGCCGTCAGAAACTACCCC
GCTTACCATGCTCCGTGTTGCCGAATTAGCATCTGAGGCAGGCATTCCGGA
CGGTGTGTTCAACGTCGTGACCGGCTCAGGTGCGGTTTGTGGTGCCGCGC
TGACTTCGCATCCACATGTAGCGAAGATCAGTTTCACCGGCTCCACAGCGA
CTGGCAAAGGCATTGCGCGTACTGCGGCGGATCACTTAACGCGCGTGACG
CTGGAACTGGGCGGAAAGAATCCGGCGATCGTCCTGAAAGATGCCGATCC
TCAGTGGGTGATTGAGGGCCTGATGACCGGGAGCTTCCTGAATCAAGGCC
AGGTCTGTGCAGCGTCTAGCCGTATCTACATTGAGGCACCGCTGTTTGACA

CCCTGGTATCAGGTTTCGAGCAAGCGGTAAAATCCTTACAAGTGGGTCCGG
GCATGTCCCCGGTAGCGCAGATTAATCCGCTGGTGAGTCGTGCACATTGCG
ACAAAGTGTGTTCGTTCCTGGATGACGCACAGGCTCAGCAAGCGGAACTG
ATCCGTGGAAGCAATGGCCCTGCTGGGGAAGGATATTACGTTGCCCCGACA
CTGGTGGTGAACCCGGATGCTAAACTGCGCTTAACCCGCGAAGAAGTCTT
TGGGCCTGTTGTTAACCTGGTTCGCGTTGCCGATGGTGAAGAGGCGCTTCA
GTTAGCCAATGACACGGAGTATGGTCTGACCGCCAGTGTATGGACGCAGA
ACCTGAGTCAGGCACTCGAATACAGCGATCGCCTGCAAGCAGGCACCGTT
TGGGTGAACTCCCACACGTTGATCGATGCTAATCTGCCCTTTGGCGGCATG
AAGCAGTCTGGCACGGGGCGCGACTTTGGACCGGATTGGCTGGATGGATG
GTGTGAAACCAAATCAGTGTGCGTACGCTATTGA

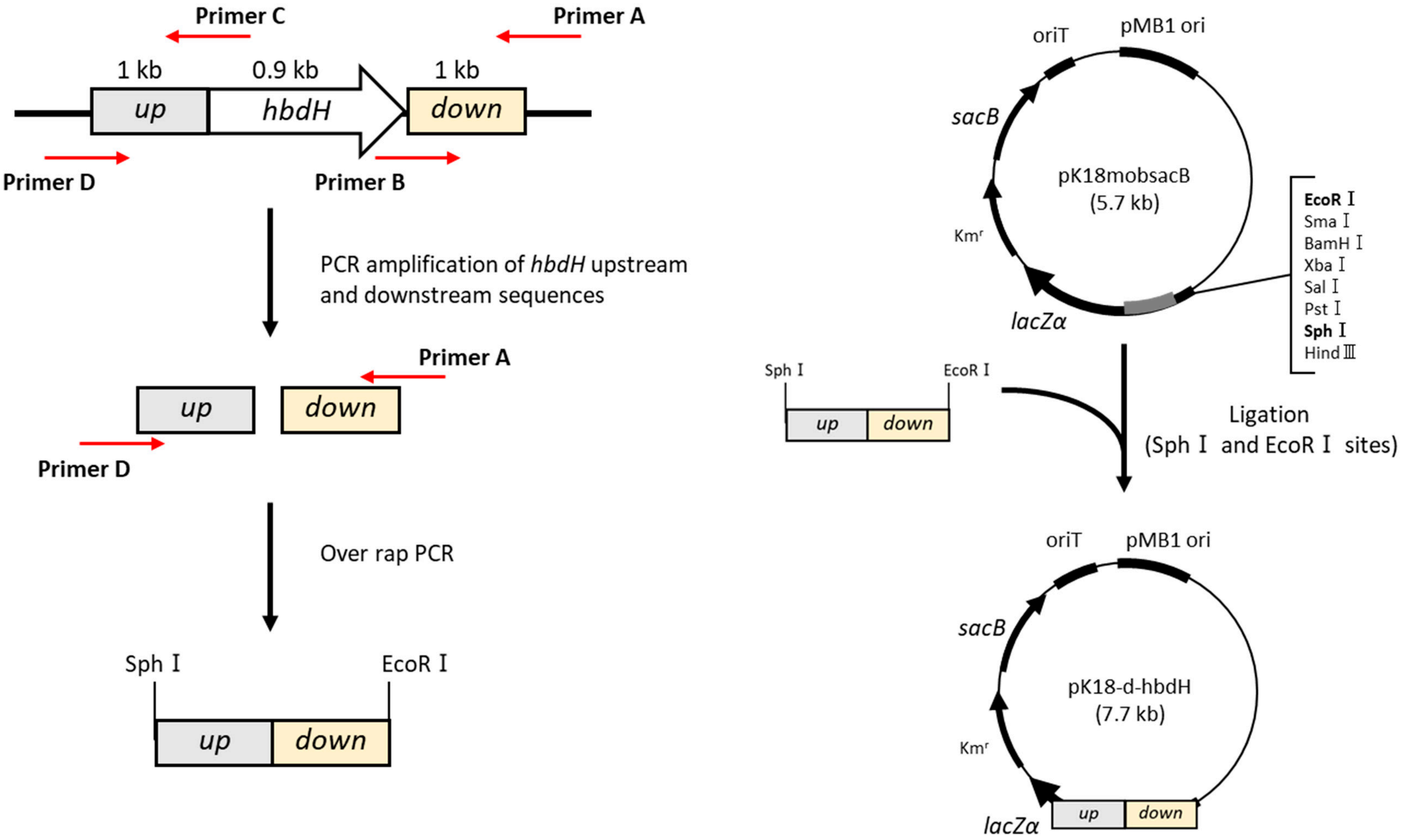


**Figure S1** Construction of the suicide plasmid of pK18-d-hbdH

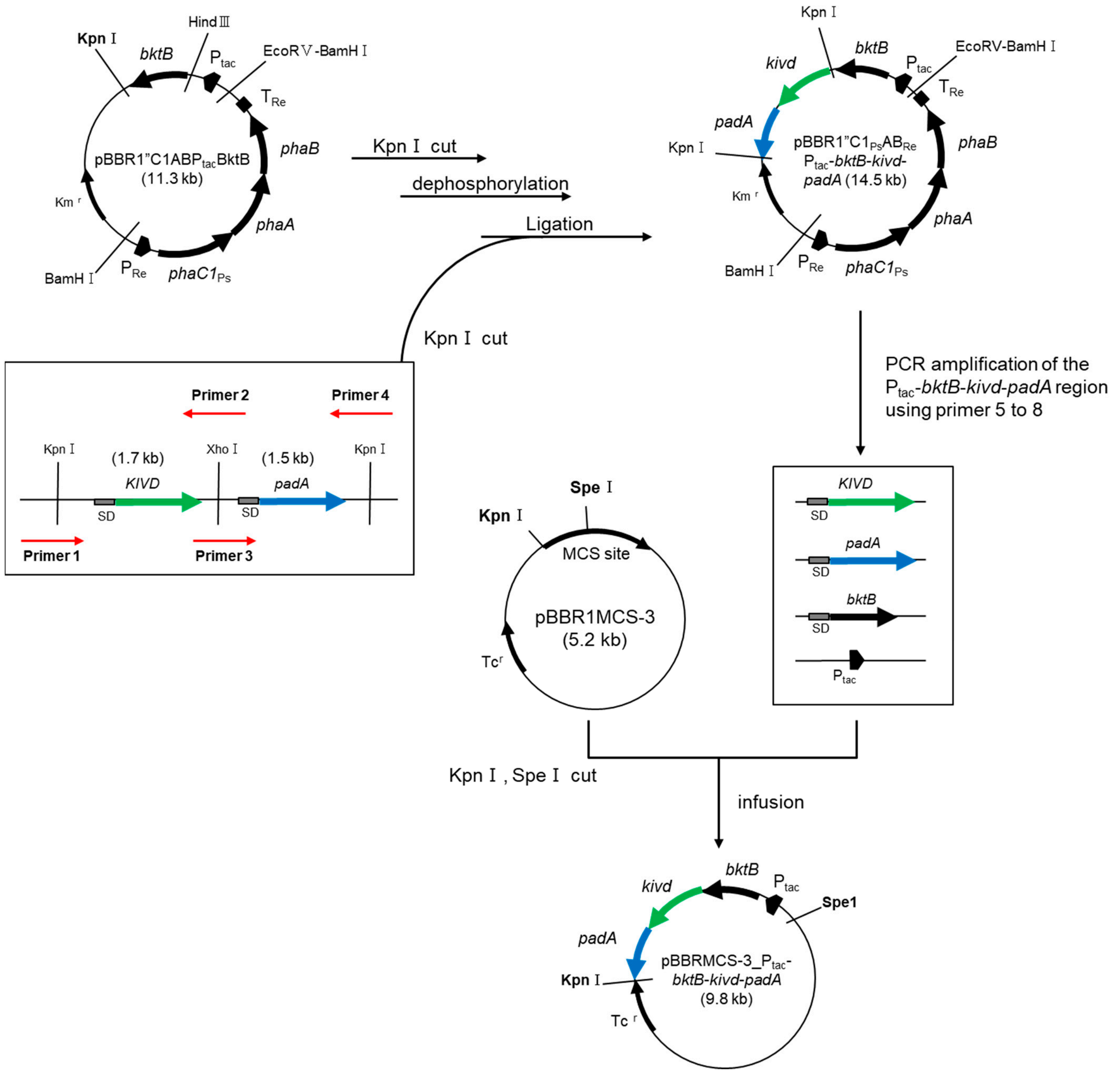


**Figure S2** Construction of pBBR1"C1$_{Ps}$AB$_{Re}$P$_{tac}$-*bktB-kivd-padA* and pBBRMCS-3_P$_{tac}$-*bktB-kivd-padA*

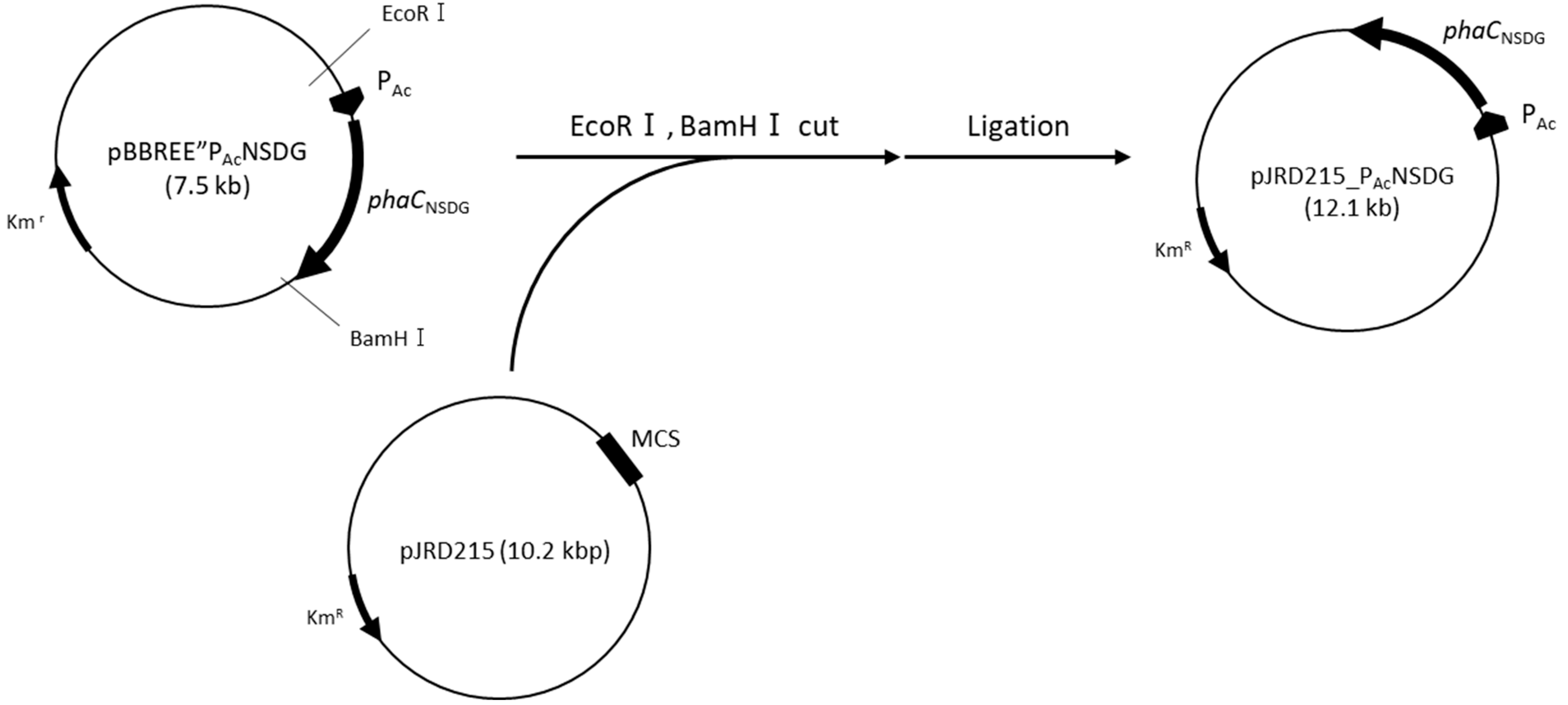


**Figure S3** Construction of pJRD215_$P_{Ac}$_*phaC*$_{Ac}$_*NSDG*
